# Greater reproductive investment, but shorter lifespan, in agrosystem than in natural-habitat toads

**DOI:** 10.7717/peerj.3791

**Published:** 2017-09-12

**Authors:** Francisco Javier Zamora-Camacho, Mar Comas

**Affiliations:** 1 Department of Biological Sciences, Dartmouth College, Hanover, NH, United States of America; 2 Department of Biogeography and Global Change, Museo Nacional de Ciencias Naturales (MNCN), Spanish National Research Council (CSIC), Madrid, Spain; 3 Estación Biológica de Doñana (EBD), Spanish National Research Council (CSIC), Sevilla, Spain

**Keywords:** Amphibian declines, Conservation, Habitat anthropization, Life-history theory

## Abstract

Global amphibian decline is due to several factors: habitat loss, anthropization, pollution, emerging diseases, and global warming. Amphibians, with complex life cycles, are particularly susceptible to habitat alterations, and their survival may be impaired in anthropized habitats. Increased mortality is a well-known consequence of anthropization. Life-history theory predicts higher reproductive investment when mortality is increased. In this work, we compared age, body size, and different indicators of reproductive investment, as well as prey availability, in natterjack toads (*Epidalea calamita*) from agrosystems and adjacent natural pine groves in Southwestern Spain. Mean age was lower in agrosystems than in pine groves, possibly as a consequence of increased mortality due to agrosystem environmental stressors. Remarkably, agrosystem toads were larger despite being younger, suggesting accelerated growth rate. Although we detected no differences in prey availability between habitats, artificial irrigation could shorten aestivation in agrosystems, thus increasing energy trade. Moreover, agrosystem toads exhibited increased indicators of reproductive investment. In the light of life-history theory, agrosystem toads might compensate for lesser reproductive events—due to shorter lives—with a higher reproductive investment in each attempt. Our results show that agrosystems may alter demography, which may have complex consequences on both individual fitness and population stability.

## Introduction

Anthropic alterations of the environment are triggering a global change in our planet, affecting ecological interactions in complex ways (Vitousek, 1994). Along with climate warming (IPCC, 2007), land use change is one of the capital drivers of this global change (Watson et al., 2000). The main causes of land use change are the massive shift from extensive to intensive agriculture (Nonhebel, 2002), and the escalation in global agriculture land surface (Veldkamp & Lambin, 2001). These changes in agriculture land use may have a major impact on wildlife (Hamilton et al., 2015).

Agrosystems can support a considerable plant (Valencia et al., 2014) and animal biodiversity (Durán, Duffy & Gaston, 2014). Nevertheless, agrosystems pose several threat factors for the biota (Green et al., 2005). Firstly, landscape alterations may make habitat inappropriate for extant species, or favor incoming ones, giving rise to complex, artificial ecological relationships (Gulinck, 1986). The plant species raised are artificially favored, impeding the system to reach a sustainable balance (Kareiva et al., 2007). To this end, fertilizers are added to agrosystem soils, provoking eutrophication of surrounding waters (Addiscott & Thomas, 2000). Plus, most pesticides have a negative impact on target-species populations as well as on other species, decreasing diversity (Teodorescu & Cogălniceanu, 2005), which may alter ecosystem functions (McMahon et al., 2012). Human presence, which can be viewed as predation risk by many animals (Frid & Dill, 2002), is a constant in agrosystems.

Amphibians may be particularly sensitive to such habitat alterations. Most amphibians undergo complex life cycles, including an aquatic larval stage, which can be jeopardized as a result of water quality loss (Lajmanovich et al., 2010; Liu et al., 2011). Moreover, the permeable skin of amphibians may facilitate absorption of pollutants (Marco et al., 2001), which they may also ingest through invertebrates they feed on (Attademo, Peltzer & Lajmanovich, 2005). Pesticides could diminish prey availability for amphibians, which, along with the aforementioned ecological and physiological stressors in agrosystems, could restrict matter and energy transmission, and ultimately, growth rates (Madsen & Shine, 2000). Agrosystems exert negative effects on both tadpole and adult development and growth (Wood & Welch, 2015). Morphology and functioning of gonads may appear altered in agrosystem amphibians (McCoy et al., 2008). In fact, morphological abnormalities are more frequent in agrosystems than in pristine habitats (Agostini et al., 2013). Agrosystem conditions have also proven to deteriorate amphibian health (Hegde & Krishnamurthy, 2014), and increase parasite prevalence (Attademo et al., 2011; Attademo et al., 2012). Overall, agrosystems pose one of the main causes of amphibian global decline (Blaustein, Wake & Sousa, 1994; Houlahan et al., 2000; Alford, Dixon & Pechmann, 2001; Allentoft & O’Brien, 2010). Along with competition by alien species, pollution, road-kill, direct predation by humans, and emerging diseases (Hels & Buchwald, 2001; Beebee & Griffiths, 2005; Corn, 2005), the major threats for amphibians are habitat loss, destruction, and fragmentation (Cushman, 2006; Dixo et al., 2009). All of them are forecasted to increase due to an expansion of intensive agriculture land use (Godfray et al., 2010).

Human-modified environments can trigger fast and intricate adaptive changes in organism traits and fitness (Carroll et al., 2007; Carroll et al., 2014; Alberti et al., 2017; Rivas-Torres et al., 2017). Amphibians can thus adapt to anthropic habitats (Hua et al., 2015), although environmental deterioration often increases mortality (Schaub & Pradel, 2004; Collins & Kays, 2011). Life-history theory predicts higher reproductive investment as an adaptive response to increased mortality (Partridge & Harvey, 1988; Stearns, 1992). Indeed, other natural life-threatening conditions, such as elevated parasite prevalence (Blair & Webster, 2007) or predation pressure (Gordon et al., 2009; Walsh & Reznick, 2009), have proven to increase reproductive effort in several animal species.

Despite the described effects of agrosystem conditions on some aspects of amphibian ecology, little is known about their role in shaping life-history traits of amphibians. In this work, our goal is to assess whether agrosystem conditions affect life history of an amphibian—natterjack toad (*Epidalea calamita*)—by comparing body size, age, and several indicators of reproductive investment—plus prey availability—between natural habitats and agrosystems. We expect that age will be lower in agrosystem toads, while, in the light of life-history theory, their reproductive investment should be greater.

## Materials and Methods

### Study species

*Epidalea calamita* is a medium-sized (49–86 mm snout-vent length (SVL) in this study system) Bufonid toad that occurs in a variety of habitats in wide regions of Europe and western Asia (Gómez-Mestre, 2014). This species hibernates in northern and central cold climates of its distribution, but aestivates instead in its southern limit, where winters are milder but summer heat and aridity hinder activity (Gómez-Mestre, 2014). Although it can be active in rainy days, mainly during the reproductive period, this toad is primarily crepuscular and nocturnal (Gómez-Mestre, 2014). This species reproduces in small puddles: males use their forelimbs to hold females during oviposition (amplexus), have external egg-fertilization, and tadpoles can metamorphose in as short as 45 days (Gómez-Mestre, 2014). The species is a generalist predator, which can forage on a variety of invertebrates with no evidence of selectivity (except for animals too small to be attractive or too large to fit in its mouth), either by a “sit-and-wait” strategy, or by preying on animals it comes across while moving (Banks, Beebee & Denton, 1993; Boomsma & Arntzen, 1985).

### Study area

In order to compare natural habitat versus agrosystem, we sampled in natural pine grove Pinares de Cartaya (SW Spain: 37°20 ′N, 7°09′W), and the adjacent agricultural land. Elevation is around 100 m above sea level. Average linear distance between both sampling sites was less than 4 km. In this region, summers are dry and hot, inducing amphibian aestivation, while springs, winters, and particularly falls are rainy. Furthermore, winter temperatures are mild, so that amphibians do not hibernate. Since *E. calamita* breeds in very small puddles, or even in swamped ditches or ruts, reproductive spots are widespread in both habitats.

Pinares de Cartaya is an 11,000-hectarea woodland dominated by stone pine (*Pinus pinea*) trees. Although some debate exists on the autochthonous or human-introduced character of *P. pinea* forests in this area, recent evidence suggests that they could be native, and, in any case, they are known to be the dominant tree formation at least for the last 4,000 years (Martínez & Montero, 2004). An undergrowth of *Cistus ladanifer*, *Pistacea lentiscus*, and *Rosmarinus officinalis* natural shrubs completes the woodland formation. Adjacent area has traditionally been used as extensive smallholdings where diverse vegetables were cultivated. However, in the last decades, most agricultural land has shifted to regular intensive strawberry, raspberry, or orange crops, among others. Moreover, soils are often artificially irrigated during summer.

### Toad management

In total, we captured 96 active adult toads (38 females and 15 males in agrosystems, 21 females and 22 males in pine groves) by hand during rainy nights within the reproductive season of 2015 (January–April). Individuals were searched for randomly through the study area, to avoid any bias in the results. However, in agrosystems only public areas (tracks, ditches, boundaries, meadows, etc.) were sampled, since it was not possible to access private farms. Males were identified because they have purple vocal sacs in their throats and variable-extension blackish nuptial pads in forelimb fingers. For all individuals, we measured SVL with a millimeter-marked ruler, and body mass with a precision balance (model CDS–100; precision 0.01 g). Body size can be considered as an indicator of reproductive investment in both male (Wilbur, Rubenstein & Fairchild, 1978) and female anurans (Castellano et al., 2004; Prado & Haddad, 2005), and is positively related to reproductive success in both male (Tejedo, 1992a) and female (Tejedo, 1992b) *E. calamita* toads. We also measured forearm dorsoventral thickness with a digital caliper (model ECO T304B.W-1230; precision 0.01 mm). Muscle thickness is positively related to force (Freilich, Kirsner & Byrne, 1995), and thicker forearms mirror stronger muscles that increase male ability to grasp females during amplexus in anurans (Navas & James, 2007). Thus, relative forearm thickness could be considered as an indicator of reproductive investment in male amphibians (Greene & Funk, 2009). We also recorded nuptial pad extension of males, considered as the total number of phalanxes and palmar tubercles developing nuptial pads in the right forelimb. Nuptial pads are used to hold females during amplexus (Duellman & Trueb, 1986; Lynch & Blackburn, 1995), and may help a male keep a female grasped despite attempts by other males to replace him (Wells, 2007). Moreover, nuptial pads release pheromones that may attract females or induce oviposition (Thomas, Tsang & Licht, 1993; Willaert et al., 2013). Given their role in male reproductive success, nuptial pads might be considered as an additional indicator of reproductive investment in males (Orton et al., 2014).

Furthermore, all individuals were marked by clipping two toes, in order to avoid re-capture. Clipping of one or two toes does not significantly reduce survival (Grafe et al., 2011; Guimarães et al., 2014). Toes clipped were used to calculate toad age by skeletochronology technique (below). The wounds were properly disinfected (with clorohexidine) and treated immediately after clipping the toes (3M Vetbond™ Tissue adhesive no 1469; 3M, Maplewood, MN, USA). Toad management was carried out in the field a few minutes after capture and individuals were set free immediately after data were recorded. Toad capture and management was conducted according to Junta de Andalucía research permits (permit number AWG/MGD/MGM/CB) and University of Granada Ethics Committee (permit number 18-CEEA-OH-2013).

### Skeletochronology technique and age estimation

For age estimation, we evaluated phalanges by means of skeletochronology (Comas et al., 2016). Phalanx skeletochronology is a non-destructive method to estimate age based on the indeterminate growth patterns of ectotherms’ bones, with an estimated precision of one year (Comas et al., 2016). Indeed, in a population of *E. calamita* toads in Southern Spain, where circa-annual activity patterns are similar to those in this study, skeletochronology has a precision of one year in individuals whose actual age was known by means of mark-recapture (Tejedo, Reques & Esteban, 1997). Regions of fast osteogenesis in the cross-sections of the bones are bordered by a line when osteogenesis is inactive or slow (aestivation, in this study area). These lines of arrested growth—hereafter, LAGs—can be counted to estimate age (Hjernquist et al., 2012). We used estimated age as a proxy of habitat-specific lifespan, since age calculated by means of skeletochronology is commonly used as a surrogate of lifespan (Amat, Oromí & Sanuy, 2010; Oromí, Sanuy & Sinsch, 2012).

Phalanges were preserved in 70% ethanol. Later, several tests were run to estimate the time necessary for decalcification. Finally, the samples were decalcified in 3% nitric acid for 2 h and 30 min. Decalcified samples were conserved in PBS solution (phosphate-buffered saline) with sucrose for at least 48 h at 4 °C, until they were sectioned with a freezing microtome (HM500, MICROM) at the Estación Biológica de Doñana (EBD-CSIC), Seville (Spain). We obtained cross-sections of 16 μm. Later, cross-sections were stained with Harris hematoxylin for 20 min, and then the excess stain was rinsed by washing the slides in tap water for 5 min. Then, stained sections were dehydrated with an alcohol chain and were finally fixed with DPX (mounting medium for histology) and mounted on slides.

Thereafter, cross-sections were examined for the presence of LAGs using a light microscope (Leitz Dialux20) at magnifications from 50 to 125X. With a ProgresC3 camera, we took several photographs of various representative cross-sections, discarding those in which cuts were unsuitable for examining the LAGs. We selected diaphysis sections in which the size of the medullar cavity was at its minimum and that of the periosteal bone at its maximum (Comas et al., 2016).

Because inferring age from the number of LAGs requires knowing the annual number of periods of arrested growth for each year, we calibrated the technique with individuals of known age (six immature individuals which were not included in the analysis), which were within the range from 1 to 2 LAGs, as reported for this species in a low-elevation -as our case is- population (Oromí, Sanuy & Sinsch, 2012). The number of LAGs detected in the periosteal bone was independently counted twice by the same person but on different occasions, always blindly regarding the specimen identification (Comas et al., 2016). Amphibians were collected in winter and spring. Therefore, LAGs deposited during previous aestivation were discernible from the outer edge of the bone. Consequently, the outer edge of the bone was not counted as a LAG.

### Prey availability

We installed pitfalls in the field to assess prey availability in both habitats (Woodcock, 2005). Pitfalls consisted of 12-cm-deep, 6-cm-diameter cylindrical containers buried to the edge on the soil. We placed 15 pitfalls in each habitat, separated from each other by at least 5 m, to ensure sample independence (Ward, New & Yen, 2001). Pitfalls were activated four wet nights, every three or four weeks, from January to April, at dusk during toad activity. Pitfalls stayed open for eight hours, and their contents were individually preserved in vials filled with ethanol (96%). Then, we analyzed vial contents for invertebrates, with a 10 − 40 × binocular microscope. We ascribed each invertebrate to an operational taxonomic unit (OTU; Sneath & Sokal, 1962), generally Order, except for Hymenoptera, which were split into Formicidae and Non Formicidae. We recorded the dry weight of prey in each pitfall with a precision balance (model Radwag WTB 200; precision 0.001 g), after 48 h in a heater (RAYPA Drying Oven) at 60 °C. However, Formicidae were removed from the analyses, in order to avoid potential biases caused by their overabundance, since they appeared only in 11 pitfalls out of 120, but represented 67% of total prey availability. Similarly, Collembola and Acarina were removed, since toads are not likely to prey on them willingly due to their small size. After sifting samples out, a total of 62 potential prey items out of 291 were analyzed. Since this species is a generalist arthropod predator (Boomsma & Arntzen, 1985), this approach provides an appropriate indicator of actual toad feeding opportunities.

### Statistical analyses

Body size, relative forearm thickness, nuptial pad extension, and age met the criteria of residual normality and homoscedasticity, so they were analyzed with parametric statistics (Quinn & Keough, 2002). We first ran a two-factor MANOVA assessing overall correlations among age, SVL, body mass, and forearm thickness, with habitat, sex, and their interaction included as factors (Morin, 1986). Since nuptial pads only appear in males, nuptial pad extension was excluded from this MANOVA. Then, we partitioned the variance by conducting individual two-way ANOVAs to test the effect of sex, habitat, and their interaction on age, body mass, SVL, and forearm thickness. Since nuptial pad extension could only be measured in males, we used a one-way ANOVA to test habitat differences. In order to avoid errors induced by collinearity between SVL and age, we then conducted two different ANCOVAs for each variable, controlling either for SVL or for age, to control for possible effects. Prey availability and dry weight data did not fulfill the criteria of residual normality and homoscedasticity, so they were analyzed with Chi-square non-parametric tests (Siegel & Castellan, 1988). Statistical analyses were performed with the software R version 3.3.3 (R Development Core Team, 2017).

## Results

**Figure 1:**
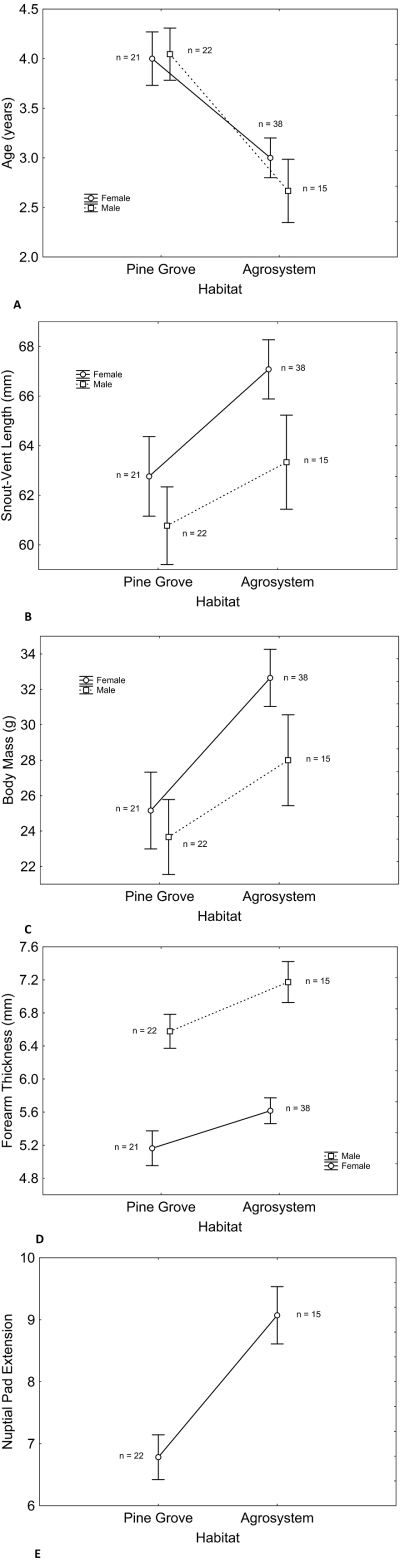
Differences in male and female toad morphology between habitats. Despite longer lifespan of pine grove toads (A) snout-vent length (B) body mass (C) forearm thickness (D) and nuptial pad extension (E) were greater in agroecosystem. Only forearm thickness was sexually dimorphic, being greater in males (D), as expected. In both habitats, sex had similar effects on all variables. Note that nuptial pads only appear in males, so no effect of sex exists. Sample sizes are indicated. Vertical bars represent standard errors.

**Figure 2:**
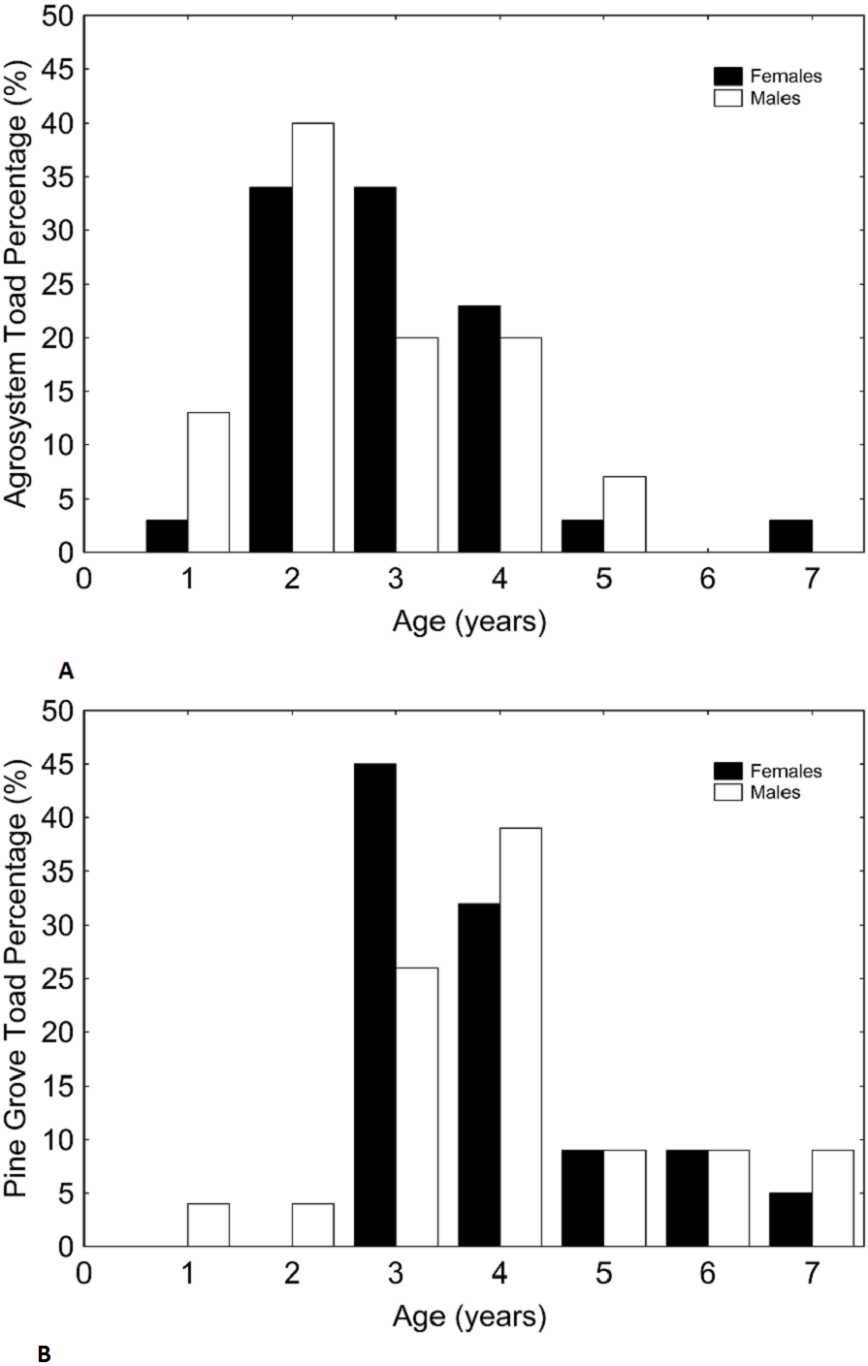
Histograms showing the distributions of percentages of male and female toad age in agrosystem (A) and pine grove (B). Black bars represent females, while white bars represent males. Sample sizes were 38 females and 15 males in agrosystem, and 21 females and 22 males in pine grove.

**Figure 3:**
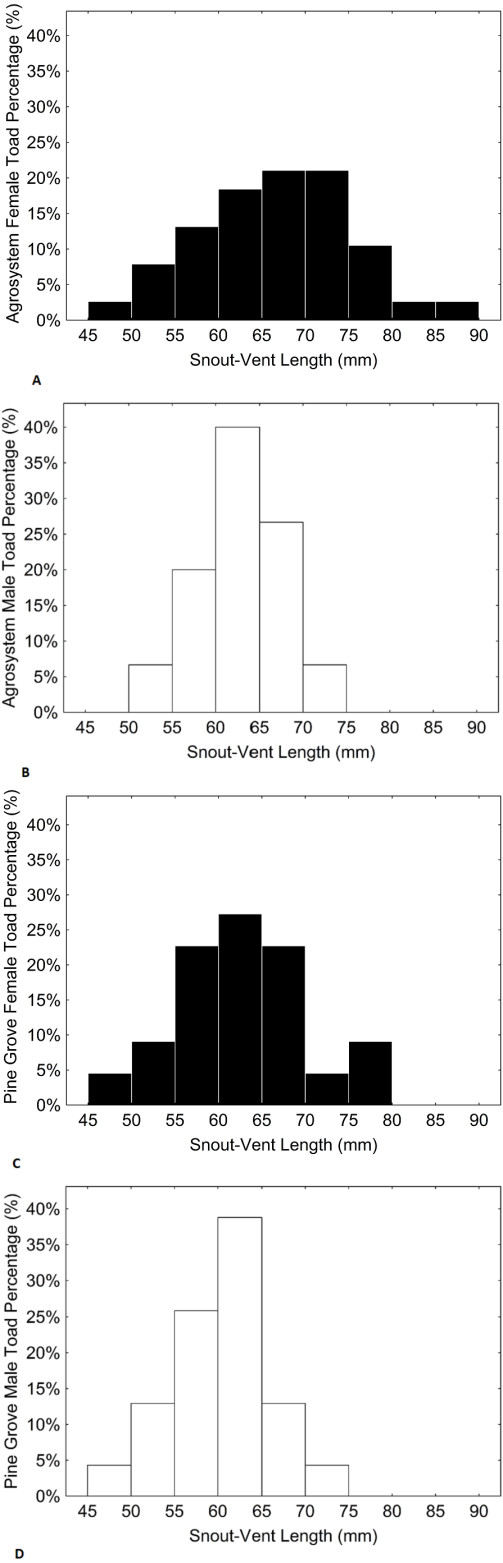
Histograms showing the distributions of percentages of male and female toad snout-vent length in agrosystem ((A) for females and (B) for males) and pine grove ((C) for females and (D) for males). Black bars represent females, while white bars represent males. Sample sizes were 38 females and 15 males in agrosystem, and 21 females and 22 males in pine grove.

**Figure 4:**
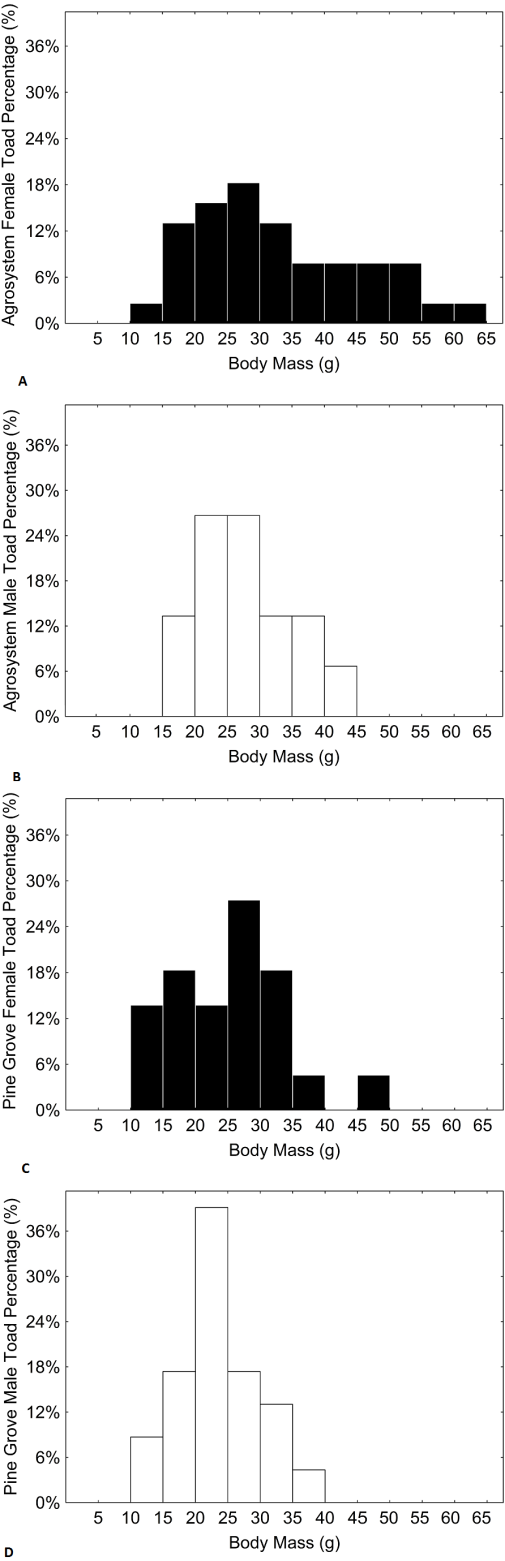
Histograms showing the distributions of percentages of male and female toad body mass in agrosystem ((A) for females and (B) for males) and pine grove ((C) for females and (D) for males). Black bars represent females, while white bars represent males. Sample sizes were 38 females and 15 males in agrosystem, and 21 females and 22 males in pine grove.

Age, SVL, body mass, and forearm thickness were correlated (Table 1). Matching with our predictions, toads showed a lower mean age in agrosystems than in pine grove (Tables 2 and 3, Figs. 1A and 2). However, SVL (Tables 2 and 3, Figs. 1B and 3) and body mass (Tables 2 and 3, Figs. 1C and 4) were greater in agrosystems toads. As for male reproductive investment indicators, both forearm thickness (Tables 2 and 3, Fig. 1D) and nuptial pad extension (Tables 2 and 3, Fig. 1E) were greater in agrosystem toads. Body mass, SVL, forearm thickness, and nuptial pad extension still differed between habitats after controlling for age, and were greater in older individuals, with the exception of nuptial pad extension, which showed no significant relationship with age (Tables 4A and 5A). However, after controlling for SVL, habitat differences disappeared for forearm thickness (Tables 4B and 5B), suggesting that thicker forearms in agrosystem might be an allometric consequence of larger body size. Nevertheless, habitat differences remained marginally non-significant for body mass, and significant for age and nuptial pad extension (Tables 4A and 5A). Except for nuptial pad extension, which showed no relationship with SVL, all variables analyzed were greater in individuals with larger SVL (Tables 4B and 5B). In all models, only forearm thickness differed between sexes, being larger in males than in females (Tables 2–5; Fig. 1D). No interaction was significant in any models. When models were rerun without non-significant interactions, results did not change qualitatively (results not shown). As for pitfall content, we found no differences in prey availability (*χ*^2^ = 2.444; *d*.*f*. = 2; *P* = 0.295) or dry weight (*χ*^2^ = 0.017; *d*.*f*. = 2; *P* = 0.898) between habitats.

**Table 1:** Summary of MANOVA results testing the effects of habitat and sex on toad age, SVL, body mass, and forearm thickness.

Source	Degrees of freedom	Wilks’ lambda	*P*-value
Habitat	4,89	0.639	<0.001
Sex	4,89	0.353	<0.001
Habitat*Sex	4,89	0.973	0.658

**Table 2:** Models testing the effects of habitat, sex, and their interaction on age, body mass, snout-vent length, forearm thickness and nuptial pad extension. Note that nuptial pads only appear in males, so there are no results involving sex. Degrees of freedom (*d.f.*) are indicated. *F*-values are shown.

Variable	*d.f.*	Habitat	Sex	Sex*Habitat
Age	1,92	19.945^***^	0.292^ns^	0.506^ns^
SVL	1,92	4.703^*^	3.270^ns^	0.307^ns^
Body mass	1,92	7.627^**^	2.057^ns^	0.543^ns^
Forearm thickness	1,92	6.400^*^	51.338^***^	0.117^ns^
Nuptial pad extension	1,35	15.220^***^		

**Notes.**

ns non-significant.

* *P* < 0.05.

** *P* < 0.01.

*** *P* < 0.001.

**Table 3:** Average values ± SE for females and males from agrosystem and pine grove of measured variables. Note that nuptial pad extension is non-dimensional, and only appears in males.

Variable	Agrosystem		Pine grove	
	Female (*n* = 38)	Male (*n* = 15)	Female (*n* = 21)	Male (*n* = 22)
Age (years)	3.00 ± 0.20	2.67 ± 0.32	4.00 ± 0.27	4.05 ± 0.26
SVL (mm)	67.08 ± 1.19	63.33 ± 1.90	62.76 ± 1.60	60.77 ± 1.57
Body mass (g)	32.64 ± 1.61	28.00 ± 2.56	25.16 ± 2.17	23.66 ± 2.12
Forearm thickness (mm)	5.62 ± 0.16	7.17 ± 0.25	5.16 ± 0.21	6.58 ± 0.20
Nuptial pad extension		9.07 ± 0.46		6.78 ± 0.36

**Table 4:** Models testing the effects of habitat, sex, and their interaction on biometrical variables, controlling for age (A) or for snout-vent length (B). Note that nuptial pads only appear in males, so there are no results involving sex. Degrees of freedom (*d.f.*) are indicated. *F*-values are shown.

(A)					
Variable	*d.f.*	Age	Habitat	Sex	Sex*Habitat
SVL	1,91	27.057^***^	19.559^***^	3.071^ns^	0.058^ns^
Body mass	1,91	22.812^***^	23.046^***^	1.753^ns^	0.216^ns^
Forearm thickness	1,91	20.023^***^	19.432^***^	65.777^***^	0.498^ns^
Nuptial pad extension	1,34	0.100^ns^	11.085^**^		

(B)					
Variable	*d.f.*	SVL	Habitat	Sex	Sex*Habitat
Age	1,91	27.057^***^	36.986^***^	0.131^ns^	0.254^ns^
Body mass	1,91	504.096^***^	3.609^§^	0.331^ns^	0.333^ns^
Forearm thickness	1,91	113.778^***^	1.767^ns^	155.770^***^	1.264^ns^
Nuptial pad extension	1,34	1.692^ns^	11.921^**^		

**Notes.**

ns non-significant.

§ marginally non-significant.

** *P* < 0.01.

*** *P* < 0.001.

**Table 5:** Average values ± SE for females and males from agrosystem and pine grove of measured variables, controlled either for age (A) or for SVL (B). Note that nuptial pad extension is non-dimensional, and only appears in males.

(A)					
Variable	Age	Agrosystem		Pine grove	
	*β*-value	Female (*n* = 38)	Male (*n* = 15)	Female (*n* = 21)	Male (*n* = 22)
SVL	0.498	68.24 ± 1.08	65.44 ± 1.72	61.07 ± 1.45	58.95 ± 1.43
Body mass	0.460	34.11 ± 1.48	30.66 ± 2.37	23.02 ± 2.00	21.36 ± 1.96
Forearm thickness	0.375	5.75 ± 0.15	7.42 ± 0.23	4.97 ± 0.20	6.37 ± 0.19
Nuptial pad extension	−0.050		9.01 ± 0.50		6.82 ± 0.38

(B)					
Variable	SVL	Agrosystem		Pine grove	
	*β*-value	Female (*n* = 38)	Male (*n* = 15)	Female (*n* = 21)	Male (*n* = 22)
Age	0.460	2.76 ± 0.18	2.73 ± 0.28	4.11 ± 0.24	4.31 ± 0.24
Body mass	0.909	28.95 ± 0.65	28.96 ± 1.01	26.83 ± 0.86	27.80 ± 0.85
Forearm thickness	0.632	5.33 ± 0.11	7.25 ± 0.17	5.29 ± 0.14	6.90 ± 0.14
Nuptial pad extension	0.189		8.94 ± 0.47		6.86 ± 0.36

## Discussion

Amphibians have complex life cycles, which make them particularly susceptible to altered ecological conditions in both aquatic and terrestrial environments (Kittusamy et al., 2014; Van Meter et al., 2014). Therefore, although agrosystems can be important refuges for amphibian populations (Kolozsvary & Swihart, 1999), they pose several threats to those populations. Our result that agrosystem *E. calamita* were younger than conspecifics in natural pine grove is aligned with these findings. A lower mean age in a given population might be a consequence of a higher mortality rate (Dempster, 1975). Thus, shorter lifespan in agrosystem toads suggests that environmental stressors in agrosystems are related to higher mortality.

Strikingly, agrosystem toads reached larger body size. Larger body size in younger individuals suggests faster growth rates, matching a trade-off between lifespan and growth, as has also been found in other vertebrates (for fish see: Lee, Monaghan & Metcalfe, 2013; for lizards: Olsson & Shine, 2002; and for mammals: Rollo, 2002). Several non-mutually-exclusive physiological pathways may underlie trade-offs between lifespan and growth rate (Metcalfe & Monaghan, 2003). Increased metabolism by a faster growth rate could lead to imbalance between pro-oxidant chemical species and anti-oxidant defenses, resulting in oxidative stress, with negative consequences on animal health (Rollo, 2002). Moreover, faster growth rates may accelerate telomere abrasion, shortening lifespans (Jennings, Ozanne & Hales, 2000). Increased growth hormone secretion can also diminish survival by diverting energy from somatic maintenance to growth (Bartke, 2005). In this sense, some agrochemicals have actually proven to alter vertebrate endocrine regulation (Nichols et al., 2010).

According to life-history theory (Cox et al., 2010), habitat differences in body size could be driven by an energy trade-off between survival and reproduction. Greater mortality, and concomitant reduced reproductive events, may favor energy diversion from somatic maintenance to reproduction in agrosystem toads, in which environmental stressors compromise survival (Reznick, Bryga & Endler, 1990). Larger body size, a consequence of faster growth rate, is indeed a costly trait that suggests greater reproductive success in both males (Wilbur, Rubenstein & Fairchild, 1978) and females (Castellano et al., 2004; Prado & Haddad, 2005) of several toad species. Specifically, a positive relationship between body size and reproductive success has been found in both male (Tejedo, 1992a) and female (Tejedo, 1992b) *E. calamita* toads. Therefore, larger body mass -even after controlling for SVL and for age- indicates greater reproductive investment in both female and male *E. calamita* agrosystem toads. Moreover, agrosystem males had thicker forearms due to their larger body size, which adds a reproductive advantage during amplexus (Greene & Funk, 2009). Likewise, male nuptial pad extension was greater in agrosystem toads. Remarkably, nuptial pad extension was the only morphological variable to show no relationship with age or SVL. This finding suggests that reproductive investment of agrosystem males was higher during their whole lifetime, which highlights the relevance of both early and sustained reproduction. Environmental pollutants may alter amphibian hormone regulation (Nichols et al., 2010), with an effect on nuptial pad morphology (Van Wyk, Pool & Leslie, 2003). Anuran nuptial pads are testosterone-induced (Epstein & Blackburn, 1997; Emerson et al., 1999) and high testosterone levels increase male reproductive success (Moore & Hopwins, 2009). Accordingly, *Bufo terrestris* males from contaminated habitats had higher testosterone levels (Hopkins, Mendonça & Congdon, 1997), suggesting that reproduction is prioritized in these human-altered conditions. Our findings match with life-history theory, which predicts that higher reproductive investment may compensate fewer reproductive events due to shorter lifespan (Stearns, 2000).

On the other hand, but not mutually-exclusive, the trade-off between lifespan and growth rate in this system may have an energetic basis, since animals with high energy intake may accelerate metabolism and growth, while those with a restricted energy budget may allocate greater proportions of resources to somatic preservation to the detriment of growth (Hou, 2013). In such energy-limiting situations, fast growth rates may be prohibitive, whereas longer lives may increase the chances of reproductive success (Holliday, 1989). In fact, longer lives are a well-documented consequence of calorie restriction in several taxa (review in Giusi & Mirisola, 2015). Hence, lower food intake could explain reduced growth and older average age in pine grove (Fontana, Partridge & Longo, 2010). However, prey availability did not differ between habitats in this study system, so we found no evidence of a dietary cause of size divergence. Nevertheless, artificial irrigation during the summer (rather dry and prolonged in the study area) could shorten aestivation in agrosystems, increasing activity time available for feeding and growing. In fact, amphibians show a global trend to live longer at high elevations, probably because colder climates shrink activity periods, reducing energy intake (Zhang & Lu, 2012). Therefore, shorter activity periods in pine grove toads could limit energy budget, increasing individual age, which adds reproduction chances, to the detriment of energy-consuming growth.

Broad sense habitat alterations in agrosystems are one of the main causes of amphibian global decline (Kiesecker, Blaustein & Belden, 2001). In the global change scenario, agricultural land use extension and intensity are expected to increase in the short term (Tilman et al., 2001). Therefore, a rising number of amphibian populations are bound to face stressing agrosystem environments in the near future (Murrieta-Galindo et al., 2013). The capability of amphibians to thrive in agrosystems will strongly depend on their sensitivity to concomitant stressful conditions (Wang & Murphy, 1982). However, our study suggests that insalubrious agrosystem environments may actually reduce amphibian average age and longevity, even though amphibians might be able to survive and reproduce. In the case we report here, *E. calamita* seemed to respond with increased reproductive investment, which may compensate for lesser reproductive events, preserving populations from extinction. Therefore, in spite of the detrimental effects of human alterations on amphibian populations, our study shows that some species might thrive in agrosystems by altering life-history traits. Nevertheless, physiological consequences of energy displacement from somatic maintenance to growth and reproductive investment, along with metabolic alterations caused by agrochemicals, are rather complex. Furthermore, other amphibian species may not be able to respond to ecological alterations by agrosystems, particularly if these occur suddenly, as predicted in many areas (Zabel, Putzenlechner & Mauser, 2014). Legal limitations in the use of agrochemicals that affect amphibians (Brodeur et al., 2014), or the preventive application of buffer land strips between their application zone and wetlands where amphibians reproduce (Wagner et al., 2015) could improve amphibian populations viability in agrosystems.

## Conclusions

In conclusion, agrosystem *E. calamita* toads show a younger mean age than adjacent natural pine grove conspecifics. Strikingly, agrosystem toads had larger body sizes despite being younger, suggesting a trade-off between somatic maintenance and growth. On the other hand, agrosystem toads showed increased indicators of reproductive investment with respect to pine grove toads. In the light of life-history theory, this finding suggests that agrosystem toads might compensate for less reproductive events with a higher reproductive investment in each attempt. Our findings point out that some amphibians can withstand agrosystem conditions, but life-history and physiology are altered as a result, with complex consequences for both individual fitness and population stability.

## Additional Information and Declarations

### Field Study Permissions

The following information was supplied relating to field study approvals (i.e., approving body and any reference numbers): Field experiments were approved by Junta de Andalucía (Permit reference AWG/MGD/MGM/CB).

### Data Availability

The following information was supplied regarding data availability: Zamora camacho, Francisco javier (2017): Shorter lifespan, but greater body size and reproductive investment in agrosystem than in natural habitat in a toad. figshare.
https://doi.org/10.6084/m9.figshare.4818790.v1.
